# Pyroptosis as a candidate therapeutic target for Alzheimer’s disease

**DOI:** 10.3389/fnagi.2022.996646

**Published:** 2022-09-15

**Authors:** Yuehua Huang, Xiaoyu Li, Guifei Luo, Junli Wang, Ranhui Li, Chuyi Zhou, Teng Wan, Fenglian Yang

**Affiliations:** ^1^Department of Reproductive Medicine, Affiliated Hospital of Youjiang Medical University for Nationalities, Baise, Guangxi, China; ^2^Reproductive Medicine, Guangxi Medical and Health Key Discipline Construction Project of the Affiliated Hospital of Youjiang Medical University for Nationalities, Baise ,Guangxi, China; ^3^Hengyang Medical College, University of South China, Hengyang, Hunan, China; ^4^Industrial College of Biomedicine and Health Industry, Youjiang Medical University for Nationalities, Baise, Guangxi, China

**Keywords:** Alzheimer’s disease, pyroptosis, amyloid β, tau, neuroinflammation

## Abstract

Pyroptosis is a form of cell death mediated by inflammasomes and gasdermins, and the relevance of pyroptosis to neurodegenerative diseases is currently receiving increasing attention. Alzheimer’s disease (AD) is a chronic progressive neurodegenerative disease that is closely associated with neuroinflammation. Its main pathological features include β-amyloid (Aβ) deposition, Tau protein hyperphosphorylation and neuronal loss. Aβ, tau-induced microglia pyroptosis and polarization leading to neuroinflammation play an important role in the pathogenesis of AD. Studying the pathogenesis and treatment of AD based on cellular pyroptosis has become a new direction in AD research. In this paper, we review the research progress of pyroptosis and will focus on the pathogenic roles of pyroptosis in AD and the role of targeted inhibition of inflammasome-dependent pyroptosis in AD treatment. These results deepen our understanding of the pathogenesis of AD and provide ideas for the development of new drugs based on the regulation of pyroptosis in AD patients.

## Introduction

Alzheimer’s disease (AD) is a common progressive neurodegenerative disease characterized by central nervous cell dysfunction and neuronal loss (Masters et al., 2015; DeTure and Dickson, 2019). The main pathological hallmarks of AD are amyloid plaque and neurofibrillary tangles (NFTS), which are usually interpreted as amyloid-β (Aβ) aggregates and highly phosphorylated tau protein deposits, respectively (Price et al., 2014; Fricker et al., 2018). In the amyloid cascade hypothesis proposed by Hardy in 1991, the sequence of pathological changes in AD is from Aβ deposition to tau aggregation and subsequent neuronal damage, emphasizing the role of Aβ and tau deposition in the progression of AD (Hardy and Allsop, 1991). However, the current study suggests that neuroinflammation and pyroptosis are also key for pathological changes in AD (De Strooper and Karran, 2016; Stephenson et al., 2018). Neuroinflammation is thought to be responsible for a variety of CNS diseases, including AD, Parkinson’s disease (PD), Multiple Sclerosis (MS), etc. (Zhang et al., 2017; Heneka et al., 2018; Feng et al., 2020). Elevated levels of inflammatory factors such as IL-1β and IL-18 are often found in central system lesions, and upregulation of these cytokines implies possible neuronal damage or death, as they trigger a severe inflammatory cascade (Allan et al., 2005; Alboni et al., 2010; Spulber and Schultzberg, 2010). The increase in the levels of these factors means that neurons may be damaged or die because they trigger a series of severe inflammatory cascades.

In the current study, aggregated Aβ and tau activation of NLRP3 (NOD-like receptor protein 3) inflammasome-mediated neuroinflammatory responses and neuronal pyroptosis were found in AD patients and animal models, whereas inhibition of this set of responses attenuated the progression of AD (Meda et al., 1995; Tan et al., 2014; Han et al., 2020b; Perea et al., 2020). This suggests that neuroinflammation and pyroptosis may be potential targets in the direction of AD therapy. While the role of neuroinflammation in the course of AD is well-established, the mechanism and mode of action of pyroptosis in AD has not been systematically elucidated (Yap et al., 2019; Onyango et al., 2021). This article systematically reviews the role of CNS cellular pyroptosis in the progression of neurodegenerative diseases, especially AD, including the cell types and mediators involved, as well as potential targeted therapeutic approaches.

## Brief introduction of pyroptosis

Pyroptosis, a term coined by Cookson and Brannan, is a new form of programmed cell death that exhibits typical features of apoptosis and necrosis. Similar to apoptosis, cellular pyroptosis causes DNA damage and is TUNEL-positive. As with necrosis, cells form transmembrane pores under septic action, releasing proinflammatory cytoplasmic contents that can eventually induce cell rupture (Xie et al., 2020). The body can use a number of mechanisms to sense intra- and extracellular “danger” signals generated by invading pathogenic microbes or tissue damage. Activated Toll-like receptors (TLRs) initiate a signaling cascade that leads to cellular activation and production of inflammatory cytokines such as tumor necrosis factor (TNF), IL-6, IL-8 and type I interferons (IFNs) (Kawai and Akira, 2007). The role of Nod like receptors (NLRs) is to recognize danger signals introduced into the host cell and some NLR proteins, such as NLR family CARD domain-containing protein (NLRP)1/3, NLRP1b, NLRC4, NAIP5, NLRP6/9 are present in the same complex and play a role in sensing bacterial toxins and secretions, nucleic acids, pathogenic crystals and denatured cellular components, and synergistically activate caspase 1, ultimately leading to cellular pyroptosis and release of the inflammatory cytokines IL-18 and IL-1β (Fink et al., 2008; Bergsbaken et al., 2009; Levy et al., 2015; Zhu et al., 2017; Liu et al., 2018). In this process, many inflammasomes form an inflammasomes complex by recruiting apoptosis-associated speck-like protein containing a caspase activation and recruitment domain (ASC) and promoting ASC polymerization into large filamentous to activate caspase-1, while some inflammasomes (e.g., NLRP1 and NLRC4) can bind directly to caspase-1 (Kesavardhana et al., 2020). Although pyroptosis has been thought to be a caspase-1-driven monocyte death pathway (canonical pathway), caspase-4, –5 and –11 were later found to effectively drive pyroptosis as well (non-canonical pathway). Inflammatory caspase-4, –5, and –11 act as direct receptors that sense pathogen-encoded molecules, such as lipopolysaccharide (LPS), and undergo self-oligomerization and proximally induced self-activation (Zanoni et al., 2016; Chu et al., 2018).

**FIGURE 1:**
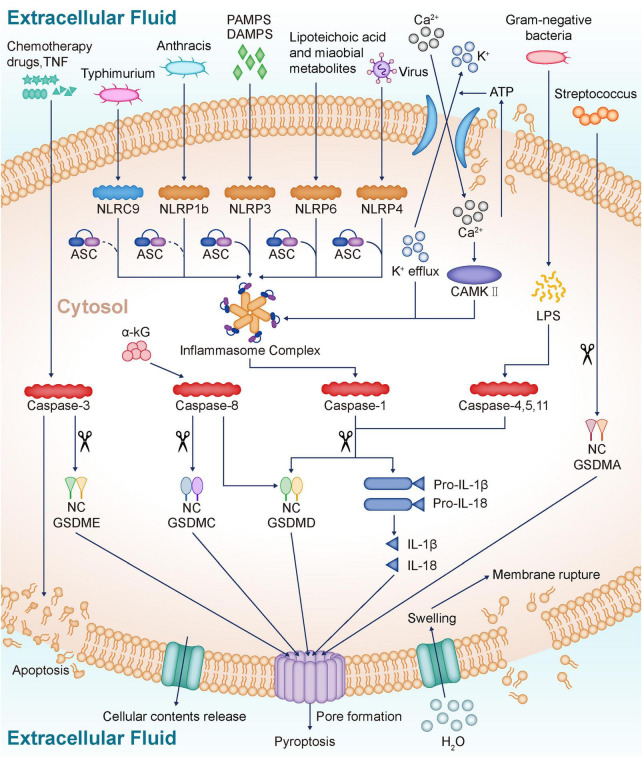
Profiles of pyroptosis. NLR proteins sense various danger signals inside and outside the cell and then activate caspase-1, which eventually leads to pyroptosis and release of inflammatory cytokines IL-18 and IL-1β. NLRC4 recognizes Typhimurium, NLRP6 recognizes lipoteichoic acid and Microbial metabolites, NLRP9 recognizes Virus, and after recruitment of ASC, eventually forms inflammasome. The inflammasome prompt caspase-1 activation, and the activated caspase-1 cleaves the GSDMD to release the GSDM N-terminal, which forms a non-selective pore in the plasma membrane through oligomerization. The formation of non-selective channels leads to membrane rupture, and IL-18 and IL-1β release, ultimately inducing pyroptosis. LPS released from gram-negative bacteria directly activates caspase-4, 5, and 11, which cleave GSDMD, ultimately leading to pyroptosis and inflammatory mediator release. α-KG induces pyroptosis *via* caspase-8-mediated GSDMC cleavage. In addition, caspase-8 also mediates the cleavage of GSDMD. Chemotherapy drugs and TNF activate caspase-3, which cleaves GSDME and induces pyroptosis. Streptococcus induces pyroptosis by direct shearing of GSDMA. ATP from damaged cells is released into the extracellular space, activating P2 × 7R, which promotes K + efflux and Ca2 + influx. The inward flow of Ca2 + activates CAMKII, which eventually activates inflammasome and induces pyroptosis. NLR, NOD-like receptors; IL-18, Interleukin-18; IL-1β, Interleukin-1β; NLRP3, NOD-like receptor protein 3; PAMPS, Pathogen-derived mediators; DAMPS, Endogenously generated mediators; NLRP1b, NOD-like receptor protein 1b; NLRC4, NOD-like receptor protein 4; NLRP6, NOD-like receptor protein 6; NLRP9, NOD-like receptor protein 9; ASC, Apoptosis-associated speck-like Protein; GSDMD, Gasdermin D; LPS, Lipopolysaccharide; α-KG, α-ketoglutarate; GSDMC, Gasdermin C; TNF, Tumor necrosis factor; GSDME, Gasdermin E; GSDMA, Gasdermin A; ATP, Adenosine triphosphate; P2 × 7R, P2 × 7 receptor; CAMKII, Calmodulin kinases II.

There are six known members of the GSDM family including GSDM-A, -B, -C -D, -E (also known as DFNB5) and DFNB59. Structurally, all GSDM members except DFNB59 have an N-terminal pore-forming structural domain, a C-terminal self-inhibitory structural domain and a loop structural domain connecting the N-terminal and C-terminal. Protease-mediated cleavage within the junctional loop can lead to the release of the GSDM N-terminal, which then forms non-selective pore channels in the plasma membrane through its oligomerization and leads to membrane rupture and subsequent pyroptosis (Zhang J. Y. et al., 2021). In 2015, gasdermin (GSDM) D was identified as a key mediator of caspase-1, –4, –5, and –11-induced pyroptosis, and these caspases activate GSDMD through protein hydrolysis, leading to pore formation and cell pyroptosis at the plasma membrane (Kayagaki et al., 2015; Shi et al., 2015). Metabolite α-ketoglutarate (α-KG) induces pyroptosis through caspase-8-mediated cleavage of Gasdermin C (GSDMC) (Zhang J. Y. et al., 2021). In addition, Caspase-8 can also mediate GSDMD cleavage and thus induce pyroptosis (Xia, 2020). Recent research have reported that streptococcus induces pyroptosis through directly shear GSDMA (Zhao et al., 2022). In addition to GSDMA, GSDMC and GSDMD, the upstream regulatory mechanisms of GSDMB, GSDME and DFNB59 still need to be further explored. The GSDMD consists of an N-terminal pore-forming structural domain, a C-terminal regulatory structural domain and a central junctional region (Ding et al., 2016; Liu et al., 2016). The GSDMD-N-terminal fragments bind to phospholipids such as phosphatidylinositol phosphate, phosphatidic acid, phosphatidylserine and cardiolipin in the inner leaflet of the plasma membrane. Then, the GSDMD-N-terminal fragments undergo sequential conformational changes, which promote their aggregation and membrane insertion, culminating in the formation of membrane pores with an inner diameter of 10–15 nm (Ruan et al., 2018; Liu et al., 2019). The formation of plasma membrane pores promotes the loss of concentration gradients, cell swelling, and ultimately cell lysis and the release of pro-inflammatory molecules and organelles such as nucleotides, IL-1 family cytokines, HMGB1, nucleic acids, mitochondria, etc. (Tsuchiya, 2021). In addition, caspase-3 proteolysis *via* Gasdermin E (GSDME) can convert tumor necrosis factor (TNF) or chemotherapy-induced apoptosis into pyroptosis (Monack et al., 1996). Adenosine triphosphate (ATP) is released into the extracellular space after cell injury and activates P2 × 7 receptor (P2 × 7R), an ATP-gated potassium channel that promotes potassium efflux while promoting calcium inward flow, which ultimately activates inflammasome-induced pyroptosis by activating Calmodulin kinases II (CAMKII) (Ratsimandresy et al., 2013; Kesavardhana et al., 2020). These will further promote the onset of cellular pyroptosis (Figure 1).

## Research progress in relationship between pyroptosis and Alzheimer’s disease

### Pyroptosis mediates Aβ/tau-induced brain injury

The onset and progression of AD is closely related to neuronal pyroptosis. Aβ can induce potassium efflux, which leads to intracellular hypokalemia and ultimately to NLRP3-mediated neuronal pyroptosis (La Rosa et al., 2019). Inflammasomes such as NLRP1, NLRP3, NLRC4, AIM2, and Pyrin can induce cell pyroptosis by activating caspase-1 (Man et al., 2017). A recent study summarized the role of NLRP1 and NLRP3 in AD pathogenesis. However, the relationship between pyroptosis and AD induced by NLRC4, AIM2 and Pyrin needs to be further elucidated (de Brito Toscano et al., 2021). Tau tangle and Aβ plaque are widely recognized as major pathogenic pathogens in AD, and tau and Aβ protein-induced pyroptosis and neuroinflammation are closely associated with AD-related brain damage (Tiwari et al., 2019). Aβ1-42 induces pyroptosis in mice cortical neurons (MCNs), increases cell permeability and promotes lactate dehydrogenase (LDH) release (Han et al., 2020b). It has been shown that Aβ activates the NLRP3-caspase-1-GSDMD axis to induce neuronal pyroptosis, thereby promoting neuroinflammatory responses and neuronal damage, and ultimately leading to accelerated progression of AD (Bai et al., 2021). In APPswe/PS1dE9 transgenic mice, NLRP1 levels were found to be upregulated in the brain. In cultured cortical neurons *in vitro*, Aβ can lead to NLRP1-mediated caspase-1-dependent neuronal pyroptosis. In contrast, *in vivo* injection of non-viral siRNA knockdown of NLRP1 or caspase-1 significantly attenuated neuronal pyroptosis and cognitive dysfunction in mice (Tan et al., 2014).

Intracerebroventricular injections of forskolin (FSK, a PKA activator) and streptozotocin (STZ) significantly increased the levels of hyperphosphorylated tau protein and pyroptosis-related proteins in mouse brain. It was found that the use of caspase-1 inhibitors, caspase-1 siRNA, or neutralizing IL-1β/IL-18 antibodies significantly attenuated FSK and STZ-induced PC12 cell injury and cognitive dysfunction in rats (Li Y. et al., 2020). Furthermore, clinical studies have shown that GSDMD, T-tau, and Tau181p levels are increased in the cerebrospinal fluid of AD patients relative to normal population and VD (vascular dementia) patients, while the levels of Aβ1-42 are decreased (Shen et al., 2021). This suggests that molecules related to the pyroptosis signaling pathway may be one of the important markers for the diagnosis and differentiation of AD. However, the molecular mechanism of tau-induced neuronal pyroptosis initiation still needs further studies to be elucidated at present (Table 1).

**TABLE 1:** AD-related injuries mediated by pyroptosis.

Experimental model	Pathogenic proteins	Mechanism	Significance	References
AD mice	Aβ	Activating NLRP3-caspase-1-GSDMD axis and inducing neuronal pyroptosis	Promoting neuroinflammation and neuronal injury, leading to accelerated progression of AD	Han et al., 2020b
AD mice	Aβ	Activating NLRP1-caspase-1-GSDND axis and inducing neuronal pyroptosis	Leading to brain damage, promoting the occurrence and development of AD	Tan et al., 2014
AD mice	Aβ	Activating NLRP3-caspase-1-GSDMD axis and inducing neuronal pyroptosis	Improving neuronal pyroptosis and playing a neuroprotective role	Bai et al., 2021
AD mice	Aβ, ASC-Aβ	Forming ASC-Aβ complex with ASC, activating NLRP3-caspase-1-GSDMD axis	Enhancing the proinflammatory response, leading to microglia pyroptosis and the release of functional ASC, leading to a vicious cycle	Friker et al., 2020
AD-HNNs	Aβ	Activating NF-κB- miR-146a-5p - TIGAR pathway	Promoting oxidative stress and pyroptosis, accelerating the progression of AD	Lei et al., 2021
AD mice	p-tau protein	/	Promoting neuronal damage, leading to cognitive dysfunction	Li Y. et al., 2020
AD patients	p-tau protein	/	Suggesting pyroptosis signaling pathway related molecules may be one of the important markers for the diagnosis and differentiation of AD	Shen et al., 2021

### Microglia pyroptosis and polarization synergistically contribute to neuroinflammation

Microglia are intrinsic immune cells of the central nervous system with phagocytic clearance, neuromodulation and regulation of the neurological environment (Hickman et al., 2018). Under pathological conditions, microglia are closely associated with neurodegenerative lesions (Hickman et al., 2018). Under healthy conditions, the balance of Aβ and tau deposition and clearance are maintained by brain-resident microglia. However, in the brains of AD patients, this balance is disrupted. ASC (the apoptosis-associated speck-like protein containing a CARD) released due to cellular pyroptosis can be built into neighboring microglia NLRP3 (NOD-like receptor protein 3) inflammasome. ASC can also form the complex ASC-Aβ with Aβ, which not only promotes microglial pyroptosis, but also hinders the clearance of bound Aβ. Pyroptotic cells will further release ASC and inflammatory factors (Friker et al., 2020).

Microglia are the resident intrinsic immune cells of the CNS and play an important regulatory role in the maintenance of the homeostasis of the CNS internal environment. Typically, microglia can be classified into a pro-inflammatory M1 phenotype and an anti-inflammatory M2 phenotype that exert neurotoxic and neuroprotective effects, respectively (Gao et al., 2022). Studies have shown a strong link between microglia polarization and multiple neurodegenerative diseases such as Parkinson’s and Alzheimer’s disease (Lu et al., 2017; Zhang et al., 2022). It was shown that increased tau oligomers and Aβ plaque inhibit immunophagocytosis of M2 microglia and promote M1 polarization of microglia, thereby inducing sustained neuroinflammation (Tang and Le, 2016). Sustained neuroinflammation will lead to BBB damage, neuronal injury, glial cell activation and BACE (Beta-Secretase) upregulation, ultimately promoting intercellular Aβ production (Leng and Edison, 2021). Aβ and tau induced cellular pyroptosis and microglia M1 polarization may be associated with nuclear factor kappa B (NF-κB) activation. In AD patients and AD-HHNs (human hippocampal neurons), the expression of NF-κB and miR-146a-5p (MicroRNAs) was increased, while the expression of TIGAR (TP53-induced glycolysis and apoptosis regulator) was significantly reduced. In AD-HNNs experiments, NF-κB activation was found to promote miR-146a-5p expression, which in turn downregulated TIGAR expression, ultimately leading to oxidative stress and promoting neuronal pyroptosis (Lei et al., 2021). Thus, Aβ- and tau-induced microglia pyroptosis and M1 polarization promote each other, ultimately inhibiting the catabolism of Aβ and tau and worsening inflammatory damage. Promoting microglia M2 polarization and inhibiting pyroptosis-related signaling pathways may become an effective therapeutic strategy. In the LPS-stimulated HFFD (high fat/fructose diet) AD rat model, Palonosetron (5-hydroxytryptamine 3 receptor inhibitor) or Methyllycaconitine (Alpha7 Nicotinic Acetylcholine Receptor inhibitor) treatment inhibited expression of glial fibrillary acidic protein in the hippocampus and promotes M2 polarization in microglia. In addition, these regimens reduced ASC expression and inhibited the activation of caspase-11, caspase-1, IL-1β and IL-18. Thus, Palonosetron/Methyllycaconitine may inhibit the progression of AD by suppressing pyroptosis and microglia M1 polarization (Mohamed et al., 2021).

### Peripheral pyroptosis plays a promotive role in Alzheimer’s disease progression

Pyroptosis can mediate both microbially induced inflammation and aseptic inflammation, which may be beneficial or pathological (Fischer et al., 2021). Recent data suggest that both mammalian pyroptosis-associated caspases are closely associated with the regulation of inflammation and immunity. In a specific cellular context, pathogen-infected cell pyroptosis can inhibit further tissue destruction and the development of chronic inflammation, whereas common cellular pyroptosis will induce not only loss of functional cells, but also focal inflammatory reaction (Songane et al., 2018; Wang et al., 2020). In addition to neuroinflammation, systemic inflammation is also involved in a variety of neurodegenerative pathologies, such as PD and AD (La Vitola et al., 2021; Rossi et al., 2021). In PBMCs (peripheral blood mononuclear cells) of patients with AD and aMCI (amnestic mild cognitive impairment), the typical inflammasomes NLRP3/caspase-1/GSDMD signaling pathway-mediated pyroptosis is activated. It was found that plasma IL-1β levels were significantly elevated in AD and aMCI and patients relative to normal controls. Patients’ inflammatory plasma IL-1β levels and Aβ1-42 levels in CSF (cerebrospinal fluid) were negatively correlated with MMSE (Mini Mental State Examination) and MoCA (the Montreal Cognitive Assessment) scores. In addition, *in vivo* experiments in mice showed that peripheral inflammasomes-induced pyroptosis can worsen neuroinflammation and thus aggravate the pathophysiological process of AD (Rui et al., 2021).

### Pyroptosis is an emerging target for Alzheimer’s disease treatment

#### Aβ/tau-induced pyroptosis is a potential target for Alzheimer’s disease treatment

Targeted inhibition of inflammasomes-dependent pyroptosis was found to improve AD-related symptoms. *In vitro*, pretreatment with MCC950 (a specific NLRP3 inhibitor) ameliorated Aβ1-42 stimulation-induced pyroptosis in human primary neurons (HPNs), thereby significantly reducing the neurotoxicity of Aβ1-42. *In vivo*, intervention with MCC950 significantly improved spatial memory capacity and brain histomorphology in SAMP8 mice and reduced Aβ deposition in the brain (Li J. et al., 2020). SH (sodium houttuyfonate) inhibited the NLRP3/GSDMD pathway and improved hippocampal neuronal pyroptosis and spatial learning memory deficits in Aβ1-42-induced AD mice (Zhao et al., 2021). In mouse and cellular models, AF (amentoflavone) downregulated NLRP3 and other pyroptosis-related protein levels through activation of AMP-activated protein kinase (AMPK)/Glycogen synthase kinase-3beta (GSK3β) signaling pathway, ultimately inhibiting Aβ1-42-induced hippocampal neuronal pyroptosis (Zhao et al., 2019). L7 (N-salicyloyl tryptamine derivatives) antagonizes Aβ-induced pyroptosis in BV2 cells by inhibiting NLRP3-Caspase-1 signaling pathway and thus exerts neuroprotective effects by down-regulating GSDMD expression (Bai et al., 2021). In the Amyloid precursor protein (APP)/presenilin-1 (PS1) mouse model, U50488H (κ-opioid receptor agonist) ameliorated synaptic plasticity and AD-related symptoms by inhibiting NLRP3-induced hippocampal microglia pyroptosis (Song et al., 2021). With the advancement of technology, more and more AD-related risk regulatory elements and genes are being identified (Novikova et al., 2021). It was found that DJ-1 protein protects dopaminergic neurons and inhibits neurodegeneration by inhibiting ROS production, and DJ-1 is therefore considered a protective factor that promotes neuronal cell survival (Dolgacheva et al., 2019). It was found that DJ-1 gene overexpression significantly ameliorated brain damage, Aβ deposition and cognitive dysfunction in 5XFAD transgenic mice. In AD mouse models, DJ-1 promotes nuclear translocation of nuclear factor erythroid 2-related factor 2 (NRF2) protein, which exerts antioxidant effects (Cheng and Zhang, 2021). In addition, DJ-1 may also reduce hippocampal neuronal pyroptosis by inhibiting caspase-1 expression and reducing reactive oxygen species (ROS) -induced NLRP3 activation (Liu et al., 2017; Cheng and Zhang, 2021). Targeted inhibition of NLRP1, caspase-1 and caspase-6 was shown to improve neuroinflammation and cognitive impairment in AD transgenic mice (Flores et al., 2021). Schisandrin (SCH) ameliorates cognitive dysfunction in AD mice by inhibiting NLRP1 inflammasomes-mediated neuronal pyroptosis and neuronal apoptosis (Li et al., 2021). Bushen Huoxue Acupuncture reduces Aβ production in the hippocampal tissue of SAMP8 mice, inhibits NLRP1 inflammasomes activation-mediated pyroptosis, and ultimately improves learning memory impairment in AD mice (Zhang T. et al., 2021).

Targeted inhibition of caspase-GSDM-dependent pyroptosis exerted significant neuroprotective effects in experiments with animal models of AD. Lithium chloride (LiCl) inhibited phosphorylated tau protein levels while also significantly reducing caspase-1 activity and inflammatory factors in forskolin (FSK) or streptozotocin (STZ) treated PC12 cells (Li Y. et al., 2020). In APP/PS1 mice, mafenide (MAF) derivatives inhibited GSDMD activation-induced pyroptosis and neuroinflammation by inhibiting GSDMD-Asp275 site cleavage (Esmaeili-Mahani et al., 2021).

#### Maintaining blood-brain barrier integrity by pyroptosis suppression reduced Aβ aggregation

The blood-brain barrier (BBB), a physical and biochemical barrier, plays a fundamental role in regulating blood flow and maintaining the homeostatic microenvironment of the central nervous system (CNS) (Ye et al., 2022). Studies have shown that inflammatory factors released from pyroptotic neuronal cells in cerebrovascular disease severely compromise the blood-brain barrier integrity (Ye et al., 2022). The negative regulatory effect of pyroptosis on the structure and function of the BBB has attracted the attention of researchers. BBB and lymphatic system dysfunction are closely associated with the accumulation of Aβ in the brain and the development of post-stroke AD (Lyu et al., 2021; Wan et al., 2022). It was shown that YZFDF (Yi-Zhi-Fang-Dai formula) significantly attenuated neurological deficits and cerebral infarction after brain I/R in rats. YZFDF inhibited glial cell pyroptosis-induced blood-brain barrier collapse and water channel protein 4 depolarization by inhibiting caspase1/11 activation and Gasdermin D cleavage, which ultimately reduced Aβ acute accumulation and formation of Aβ1-42 oligomers (Lyu et al., 2021). Traumatic brain injury (TBI) induces NLRP3 inflammasome-mediated pyroptosis of damaged BMVECs (brain micro-vascular endothelial cells). caspase-1 inhibitor AcYVAD-CMK can inhibit this process by blocking GSDMD cleavage and ASC oligomerization to inhibit this process, thereby maintaining blood-brain barrier integrity (Liu et al., 2022).

#### Non-coding RNA can be a tool for pyroptosis inhibition in Alzheimer’s disease

**FIGURE 2:**
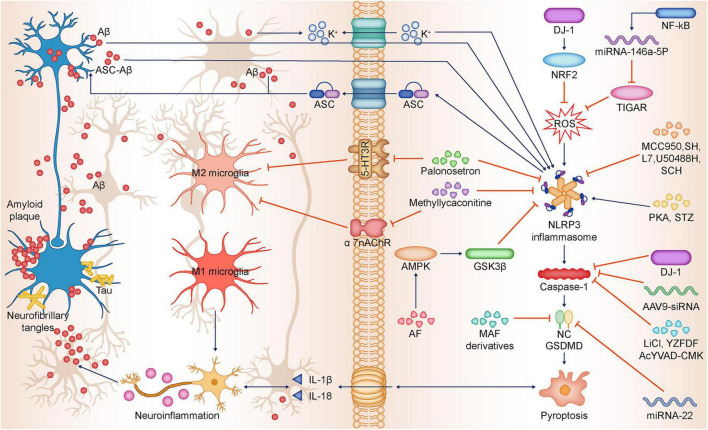
Relationship between NLRP3-dependent pyroptosis and Alzheimer’s disease. Aβ deposition and Tau hyperphosphorylation are the main pathological features of AD, and Aβ and Tau in turn can induce neuronal pyroptosis and neuroinflammation, thus promoting AD progression. Aβ promotes pyroptosis by activating the NLRP3-caspase-1-GSDMD axis. Besides Aβ promotes K + efflux, which triggers NLRP3 inflammasome activation and subsequent pyroptosis. ASC can bind to Aβ to form ASC-Aβ, which promotes pyroptosis by activating NLRP3 inflammasome. Tau and Aβ inhibit immunophagocytosis of M2 microglia, promote M1 microglia polarization, and induce sustained neuroinflammation. Palonosetron and Methyllycaconitine inhibit 5-HT3R and α7-nAChR, respectively, which promotes microglia M2 polarization while inhibiting NLRP3 inflammasome expression. NF-κB activates and promotes miR-146a-5p transcription, which inhibits TIGAR expression, ultimately leading to ROS occurrence and promoting NLRP3 activation. DJ-1 promotes nuclear translocation of NRF2 and inhibits ROS production, thereby suppressing NLRP3 activation. FSK, STZ promote NLRP3 inflammasome expression. MCC950, SH, L7, U50488H, SCH inhibit NLRP3 and attenuate neuronal pyroptosis. AF activates AMPK/GSK3β pathway, thus downregulating NLRP3 expression. LiCl, YZFDF, AcYVAD-CMK, AAV9-siRNA inhibit caspase-1 activation, thereby inhibiting neuronal pyroptosis. Mafenide derivatives, miRNA-22 inhibit GSDMD, thereby inhibiting neuronal pyroptosis. Aβ, β-amyloid; Tau, Tau protein; AD, Alzheimer’s disease; NLRP3, NLR family pyrin domain-containing 3; ASC, Apoptosis-associated speck-like Protein; ASC-Aβ, Complex ASC-Aβ; NF-κB, Nuclear factor kappa β; TIGAR, TP53-induced glycolysis and apoptosis regulator; ROS, Reactive oxygen species; DJ-1, DJ-1 gene; NRF2, Nuclear factor E2-related factor 2; FSK, Forskolin; STZ, Streptozotocin; MCC950, NLRP3 inflammasome inhibitor; SH, Sodium houttuyfonate; L7, N-salicyloyl tryptamine derivatives; U50488H, k-opioid receptor agonist; SCH, Schisandrin; AF, Amentoflavone; AMPK, AMP-activated protein kinase; GSK3β, Glycogen synthase kinase-3beta; liCl, Lithium chloride; YZFDF, Yi-Zhi-Fang-Dai formula; AcYVAD-CMK, Caspase-1 inhibitor; 5-HT3R, Serotonin 3 receptor; α7-nAChR, α7 nicotinic Ach receptor.

The use of siRNA, lncRNA and miRNA targeting to treat various neurodegenerative diseases has attracted increasing scholarly attention. siRNA (small interfering RNA) intervention with caspase-1 or GSDMD can inhibit Aβ1-42-induced pyroptosis. The expression of pyroptosis-related proteins was downregulated in the cerebral cortex and hippocampus of AD mice after injection of AAV9-siRNA (small interfering RNA inhibiting caspase-1), and behavioral impairment was alleviated (Han et al., 2020b). It was found that miRNA-22 (microRNAs) was negatively correlated with inflammatory factor expression in AD patients. miRNA-22 could inhibit glial cell pyroptosis and inflammatory cytokine release by targeting GSDMD, thereby improving cognitive performance in AD mice. In the APP/PS1 double transgenic mouse model, miRNA-22 mimic injection significantly improved the memory ability and behavior of mice. At the same time, it was found that the expression of GSDMD and P30-GSDMD and inflammatory factors in mouse brain tissue was reduced (Han et al., 2020a). Further studies showed that miRNA-22 inhibited pyroptosis by targeting GSDMD and improved memory and motor ability in AD mice by suppressing inflammatory responses. It was found that adipose-derived mesenchymal stem cells (ADMSCs)-derived exosomes carrying miRNA-22 could reduce the release of inflammatory factors by inhibiting pyroptosis (Zhai et al., 2021; Table 2; Figure 2).

**TABLE 2:** Interventions targeting pyroptosis in AD treatment.

Interventions	Experimental model	Mechanism	Significance	References
Palonosetron/ Methyllycaconitine	AD rats	decreasing the expression of ASC and inhibiting the activation of caspase-11, caspase-1, IL-1β and IL-18	Inhibiting the progression of AD by suppressing pyroptosis	Mohamed et al., 2021
MCC950	AD mice	Inhibiting NLRP3-caspase-1-GSDMD axis	Improving spatial memory ability and brain morphology, reducing the deposition of Aβ in the brain	Rui et al., 2021
SH	AD mice	Inhibiting NLRP3-caspase-1-GSDMD pathway	Improving hippocampal neuronal pyroptosis and spatial learning and memory deficits	Li J. et al., 2020
AF	AD mice	Activating AMPK-GSK3β signaling pathway	Reducing the levels of NLRP3 and other pyroptosis related proteins, inhibiting the pyroptosis of hippocampal neurons	Zhao et al., 2021
L7	AD mice	Inhibiting NLRP3-caspase-1-GSDMD signaling pathway	Antagonizing pyroptosis and reducing GSDMD expression, playing a neuroprotective role	Bai et al., 2021
U50488H	AD mice	Inhibiting Ca2 + -CaMKII-CREB signaling pathway	Inhibiting microglial pyroptosis, improving synaptic plasticity and playing a neuroprotective role in AD mice	Zhao et al., 2019
DJ-1	AD mice	Regulating the Nrf2 pathway	Inhibiting oxidative stress and pyroptosis in hippocampal neurons	Dolgacheva et al., 2019
DJ-1	AD mice	Inhibiting NLRP3-caspase-1-GSDMD signaling pathway	Reducing the pyroptosis of hippocampal neurons	Cheng and Zhang, 2021
SCH	AD mice	Inhibiting NLRP1-caspase-1-GSDMD signaling pathway	Inhibiting neuronal pyroptosis and neuronal apoptosis, improving cognitive dysfunction	Flores et al., 2021
LiCl	AD mice	Inhibiting the level of phosphorylated Tau protein and the activity of caspase-1	Inhibiting pyroptosis and neuroinflammation	Li Y. et al., 2020
MAF	AD mice	Inhibiting the activity of GSDMD	Inhibiting pyroptosis and neuroinflammation caused by GSDMD activation	Zhang T. et al., 2021
YZFDF	Cerebral I/R injury models in rats	Inhibiting NLRP3-caspase1-GSDMD signaling pathway	Inhibiting BBB collapse and aquaporin 4 depolarization induced by glial pyroptosis ultimately reducing acute Aβ accumulation and Aβ1-42 oligomer formation	Wan et al., 2022
AcYVAD-CMK	AD mice	Inhibiting NLRP3-caspase1-GSDMD signaling pathway	Inhibiting NLRP3 inflammasome-mediated pyroptosis of BMVECs and maintaining the integrity of blood-brain barrier in TBI	Lyu et al., 2021
AAV9-siRNA	AD mice	Inhibiting NLRP3-caspase1-GSDMD signaling pathway	Reducing The expression of pyroptosis related proteins in the cerebral cortex and hippocampus of AD mice, alleviating the behavioral damage	Han et al., 2020b
miRNA-22	AD mice	Inhibiting the expression of GSDMD	Decreasing the expression of GSDMD, P30-GSDMD and inflammatory cytokines in the brain tissue of mice, improving the memory ability and behavior of mice	Liu et al., 2022

## Discussion

This paper focuses on elucidating the mechanisms of pyroptosis-mediated AD pathogenesis and the progress of experimental studies on regulating pyroptosis in AD therapy. It is well-known that inflammasome NLRP1, NLRP3, NLRP4, and AIM2 can initiate pyroptosis-related downstream signals under the stimulation of various external factors. A large number of studies have now shown that the AD-associated pathological marker Aβ can induce pyroptosis-related neuronal damage by activating NLRP1 and NLRP3, which leads to AD progression. Therefore, neuronal pyroptosis may become an important pathogenic mechanism and marker of AD progression. However, the relationship between p-tau and neuronal pyroptosis are still barely reported and the specific molecular mechanisms still need to be further explored. In addition to the role of NLRP1 and NLRP3 in neuronal pyroptosis elucidated in AD models, the potential link between other pyroptosis-related inflammasomes and pathological markers of AD also deserves further research exploration. Furthermore, in addition to neurons in cortical, hippocampal and other brain regions mentioned above, abnormalities in glial cells such as microglia and astrocytes all play a key role in the pathogenesis and progression of AD (Bartels et al., 2020). Interestingly, recent studies have shown that ASC released by microglia pyroptosis binds to Aβ in the intercellular fluid to form ASC-Aβ polymers, thereby preventing phagocytic degradation of Aβ and promoting pyroptosis of neighboring cells (Venegas et al., 2017; Heneka et al., 2018; Lučiūnaitė et al., 2020). Thus, pro-inflammatory polarization and pyroptosis-induced neuroinflammation in microglia mediates Aβ-induced sustained injury. This evidence suggests a pathogenic role for non-neuronal glial cell pyroptosis in AD. More studies between pyroptosis and AD pathogenesis in the glial cell context need to be further developed. In addition to central nervous system inflammation, the extrinsic relationship between systemic inflammation and AD has been revealed by recent studies. These studies have demonstrated the potential of pyroptosis pathway-related proteins in peripheral blood mononuclear cells as markers of AD. However, the specificity of this peripheral marker remains to be evaluated given the broad association between pyroptosis and multiple neurological disorders.

Of interest is the remarkable progress in drug research targeting the Aβ/tau-induced pyroptosis-related pathway. Different drugs can not only attenuate neuronal loss by targeting cortical and hippocampal neurons, but also protect the blood-brain barrier by inhibiting glial cell and vascular endothelial cell pyroptosis, thereby inhibiting pathological Aβ aggregation in the brain interstitial fluid. As an intervention tool, a large number of non-coding RNAs show greater potential for the targeted modulation of focal death pathway proteins. However, a large number of drug studies are still limited to targeting NLRP3 or NLRP1/caspase-1/GSDMD classical pyroptosis pathway to inhibit neuronal pyroptosis in AD-related regions and thus alleviate AD symptoms. A more in-depth study of the link between pyroptosis and AD pathogenesis would be beneficial for drug development. In addition, it is promising that human clinical studies have found increased levels of pyroptosis-related proteins in the cerebrospinal fluid of AD patients and that pyroptosis regulatory targets related to AD pathogenesis have been identified in human neuronal cells. In conclusion, this study summarizes most of the current clues linking the relationship between AD and pyroptosis, and this evidence suggests that pyroptosis holds great promise and feasibility for the study of AD pathogenesis and drug development, and targeting pyroptosis will provide new opportunities for the treatment of AD.

## Author contributions

TW designed this article. YH, XL, and GL wrote the manuscript. JW and RL prepared figures. TW and FY critically revised the manuscript for important intellectual content. All authors have read and approved the final manuscript and agreed to be accountable for all aspects of this work.
